# New taxa of Camaenidae from northern Vietnam (Gastropoda, Stylommatophora)

**DOI:** 10.3897/zookeys.1280.185160

**Published:** 2026-05-29

**Authors:** Barna Páll-Gergely, Vukašin Gojšina, Ivailo Dedov

**Affiliations:** 1 Department of Water Management and Natural Ecosystems, Albert Kázmér Faculty of Agricultural and Food Sciences of Széchenyi István University, Vár 2., 9200 Mosonmagyaróvár, Hungary Institute of Biodiversity and Ecosystem Research, Bulgarian Academy of Sciences Sofia Bulgaria; 2 Department of Morphology, Systematics and Phylogeny of Animals, University of Belgrade, Faculty of Biology, Studentski trg 16, 11000, Belgrade, Serbia Department of Morphology, Systematics and Phylogeny of Animals, University of Belgrade, Faculty of Biology Belgrade Serbia; 3 Institute of Biodiversity and Ecosystem Research, Bulgarian Academy of Sciences, 2 Gagarin Street, 1113 Sofia, Bulgaria Department of Water Management and Natural Ecosystems, Albert Kázmér Faculty of Agricultural and Food Sciences of Széchenyi István University Mosonmagyaróvár Hungary

**Keywords:** Anatomy, Fansipan Mountains, new species, systematics, taxonomy

## Abstract

The following new taxa are described from northern Vietnam: *Fansipanica milae* Páll-Gergely & Dedov, **gen et sp. nov**., *Ducanhia* Páll-Gergely, **gen. nov**. (type species: *Helix balansai* Morlet, 1886), *Vinatachea porcellana* Páll-Gergely, **sp. nov**. The reproductive anatomy of all three species is described. Helix (Chloritis) gereti Bavay & Dautzenberg, 1900 is moved to *Fansipanica***gen nov**., and *Camaena delsaerdti* Thach & F. Huber, 2018 is moved to *Vinatachea*.

## Introduction

Camaenidae is one of the largest land snail families, with 2877 accepted extant species classified into 284 genera (MolluscaBase Eds. 2026). Most Southeast Asian species have been described at the end of the 19th and the beginning of the 20th centuries, and with very few exceptions, based on conchological characters only. In his Vietnamese checklist, Schileyko (2011) listed 127 species belonging to 16 genera (separated to Bradybaenidae and Camaenidae, which currently form a single family, see Bouchet et al. 2017). In the last ~ 15 years, there has been an increasing interest regarding the taxonomy of Vietnamese camaenids. Several new species and genera have been introduced (Thach 2016, 2018, 2020, 2021, 2023; Schileyko 2018; Páll-Gergely and Hunyadi 2019; Páll-Gergely and Neubert 2019; Páll-Gergely et al. 2020, 2022, 2023). Furthermore, the genus *Chalepotaxis* Ancey, 1887 has been excluded from the Camaenidae (Páll-Gergely et al. 2016), *Thaitropis* Schileyko, 2004 was synonymised with *Landouria* Godwin-Austen, 1918 (Nurinsiyah et al. 2019), and *Giardia* Ancey, 1907 was given a replacement name (*Anceyoconcha* S. Tumpeesuwan & C. Tumpeesuwan in Nahok et al. 2020). Although some newly introduced genera were described based on anatomical characters, the reproductive anatomy, which is considered essential for generic classification, is known only for a handful of species. In this paper, we describe two genera and two species new to science from northern Vietnam, providing data on their anatomy and distribution.

## Materials and methods

The material of *Fansipanica milae* Páll-Gergely & Dedov, sp. nov. was collected during surveys in northern Vietnam in 2023 and 2025. The samples were collected by hand, at the beginning of the dry season (9–23 October), on the eastern slope of Fansipan Peak (3040), in a monsoon-influenced deciduous montane forest with a dense bamboo understory, at ca 3030–3040 m. The remaining materials were obtained from the institutions indicated for each taxon.

The counting of the shell whorls (to the closest 0.25 whorl) follows Kerney and Cameron (1979: 13). We used the following equipment for photographing the shells: Canon EOS 2000d camera, Tamron SP AF 90 mm F/2.8 Di MACRO 1:1 macro objective, one camera-mounted flash, two studio flash units (BlitzBirne Mikrosat), and two additional lights. Shell sculpture was photographed via a Nikon SMZ25 digital microscope with Nikon Nis-Elements software. Imaging of *Fansipanica milae* Páll-Gergely & Dedov, sp. nov. in situ was carried out using an Olympus Tough TG-5 digital camera.

In the description of the reproductive system, we used the terms “proximal” and “distal” relative to the hepatopancreas.

### Abbreviations

**D** shell diameter

**H** shell height

**HNHM** Hungarian Natural History Museum (Budapest, Hungary)

**IBER** Institute of Biodiversity and Ecosystem Research (Bulgarian Academy of Sciences, Sofia, Bulgaria)

**IEBR** Institute of Ecology and Biological Resources (Vietnam Academy of Science and Technology, Hanoi, Vietnam)

**MNHN** Muséum National d’Histoire Naturelle (Paris, France)

**NHM, NHMUK** The Natural History Museum (London, UK)

## Taxonomic account

### Family Camaenidae Pilsbry, 1895

#### Fansipanica

Taxon classification

Animalia

StylommatophoraCamaenidae

Páll-Gergely & Dedov gen. nov.

460A3FC5-1D1F-5097-9A53-EECC9EF96CC5

https://zoobank.org/63E44915-CE3A-418B-8054-FC51A1351EF7

##### Type species.

*Fansipanica milae* sp. nov.

##### Diagnosis.

This camaenid genus is characterised by its small (~ 1 cm), globular, brownish, hairy shell. Genitalia with a long a cylindrical penis whose apical part covered by a penial sheath, a conical penial verge, a slender epiphallus (as slender as the vas deferens) and a very small, rounded flagellum.

##### Differential diagnosis.

There are several genera in continental Southeast Asia possessing hairy/scaly/pitted protoconchs and mostly hairy shells: *Bellatrachia* Schileyko, 2018, *Bouchetcamaena* Thach, 2018, *Burmochloritis* Godwin-Austen, 1920, *Chloritis* H. Beck, 1837, and *Trichochloritis* Pilsbry, 1891. They all differ from the new genus in their reproductive anatomy. Namely, *Bellatrachia* has a large, fleshy flagellum (Schileyko 2018; Páll-Gergely and Neubert 2019), *Bouchetcamaena* has a very long flagellum (Páll-Gergely et al. 2022), *Burmochloritis* has a penial caecum, a vermiform flagellum and a blind-ending organ deriving from the wall of vagina (Godwin-Austen 1920; Páll-Gergely et al. 2023), continental *Chloritis* species have a more strongly developed flagellum and lack a penial sheath (Sutcharit and Panha 2010; Páll-Gergely et al. 2020), and *Trichochloritis* has a penial caecum (Páll-Gergely and Neubert 2019). Furthermore, in all these genera the vas deferens is much thinner than the epiphallus, while in *Fansipanica* gen. nov. the vas deferens and the epiphallus have approximately the same diameter.

##### Etymology.

The new genus is named after the Fansipan Peak, the type locality of the new taxon. Genus feminine.

##### Remarks.

The key traits of this new genus are the penial sheath on the proximal (apical) part of the penis, and the epiphallus, which is as slender as the vas deferens. The epiphallus is much thicker than the vas deferens in all Camaenidae, and their boundary is clearly visible in the abrupt change of diameter (Schileyko 2003). In this new genus, however, only the small and blunt flagellum indicates the transition between the epiphallus and the vas deferens (besides differences of the inner surface, see Fig. 3).

The penial sheath is probably not homologous with that of other genera of Camaeninae, covering mostly the distal (basal) part of the penis. However, it may be homologous with the thin fibrous capsule covering the proximal part of the penis of *Bouchetcamaena platytropis* (Möllendorff, 1894) (see Páll-Gergely et al. 2022).

#### Fansipanica milae

Taxon classification

Animalia

StylommatophoraCamaenidae

Páll-Gergely & Dedov sp. nov.

A15685B3-71C8-5B05-89F4-D2F2D0EA4A36

https://zoobank.org/5ED095AF-8F3A-4886-9C76-488A00BF2774

Figs 1, 2, 3, 4

##### Type material.

***Holotype*** (D: 12.8 mm, H: 9.5 mm); • **Vietnam**, Northern, Lào Cai Province, Sa Pa district, Fansipan Peak, bamboo forest below the peak, 22.3046°N, 103.7759°E, ca 3040 m, 24 X 2023, leg. I. Dedov, N. Simov, R. Bekchiev, M. Langurov, HNHM 105552. ***Paratypes***: • IBER Coll.No.40619/2, same locality; IBER Coll.No.40633/1, same locality, 22.3052°N, 103.7756°E, ca 3033 m, 09 X 2025, leg. N. Simov, K. Ivanov.

**Figure 1.**
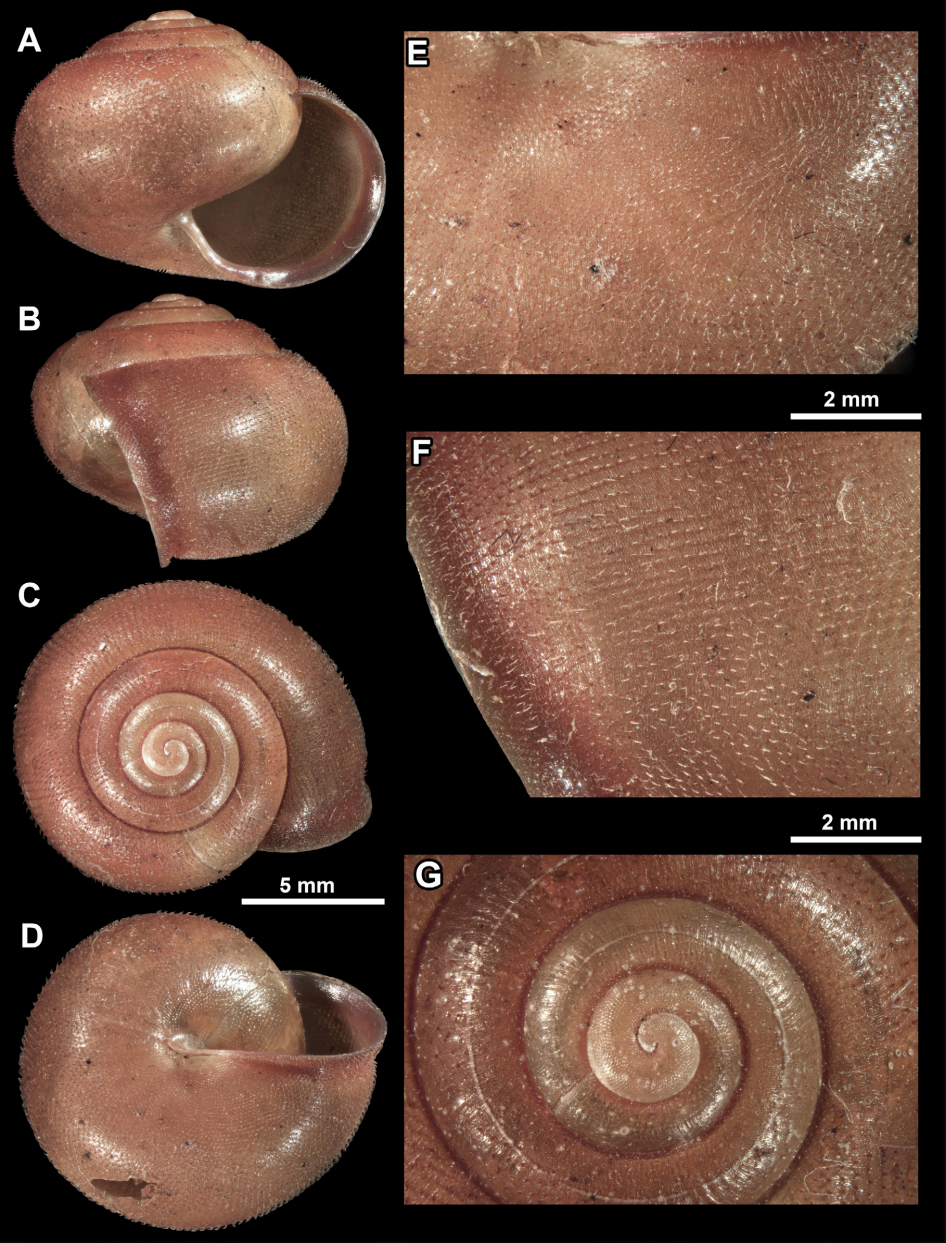
Holotype of *Fansipanica milae* gen. et sp. nov. **A**. Apertural view; **B**. Lateral view; **C**. Dorsal view; **D**. Ventral view; **E**. Sculpture of the ventral side; **F**. Sculpture of the last whorl just behind the peristome; **G**. Protoconch and first teleoconch whorls.

**Figure 2.**
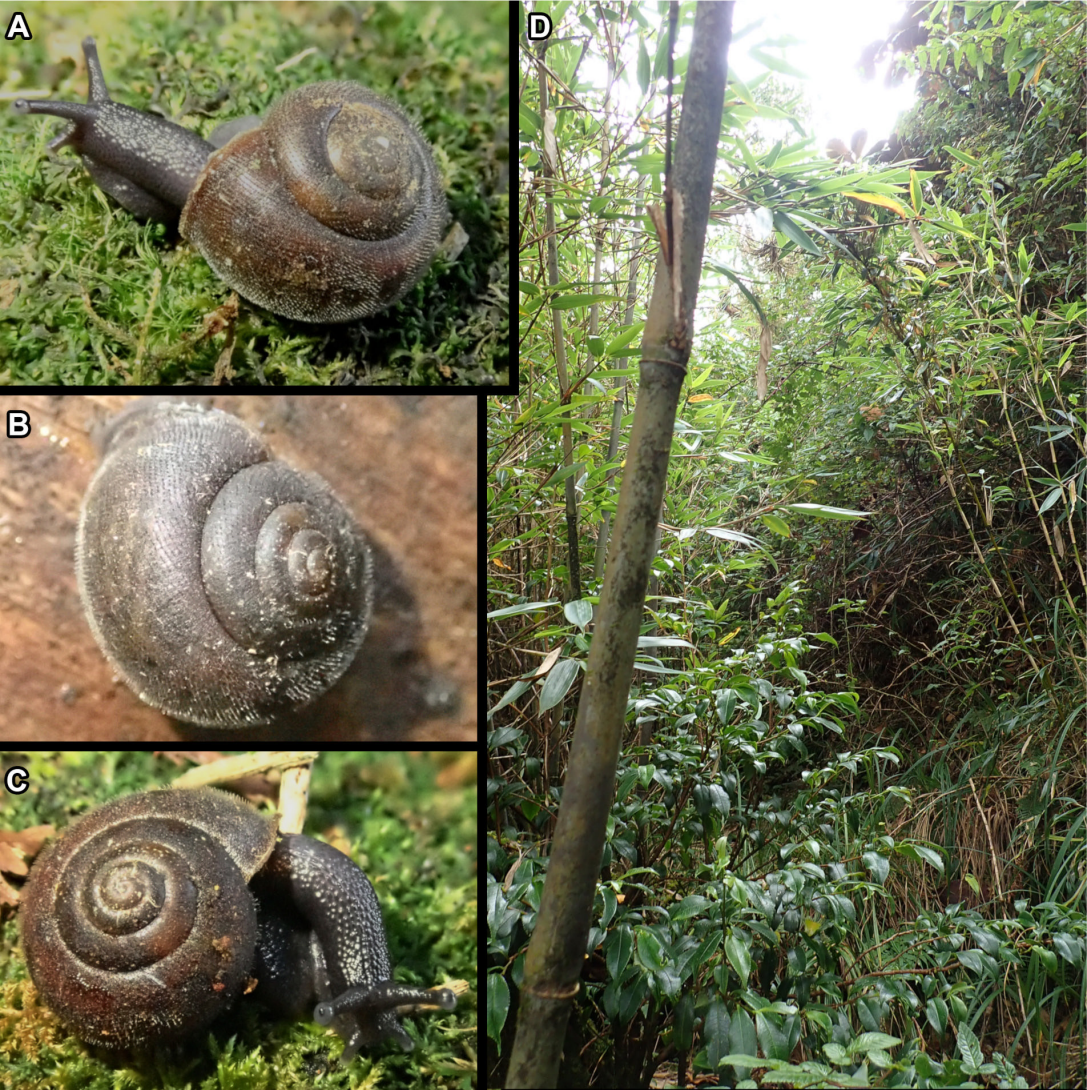
**A–C**. Living specimen *Fansipanica milae* gen. et sp. nov. **D**. Habitat of *Fansipanica milae* gen. et sp. nov.

**Figure 3.**
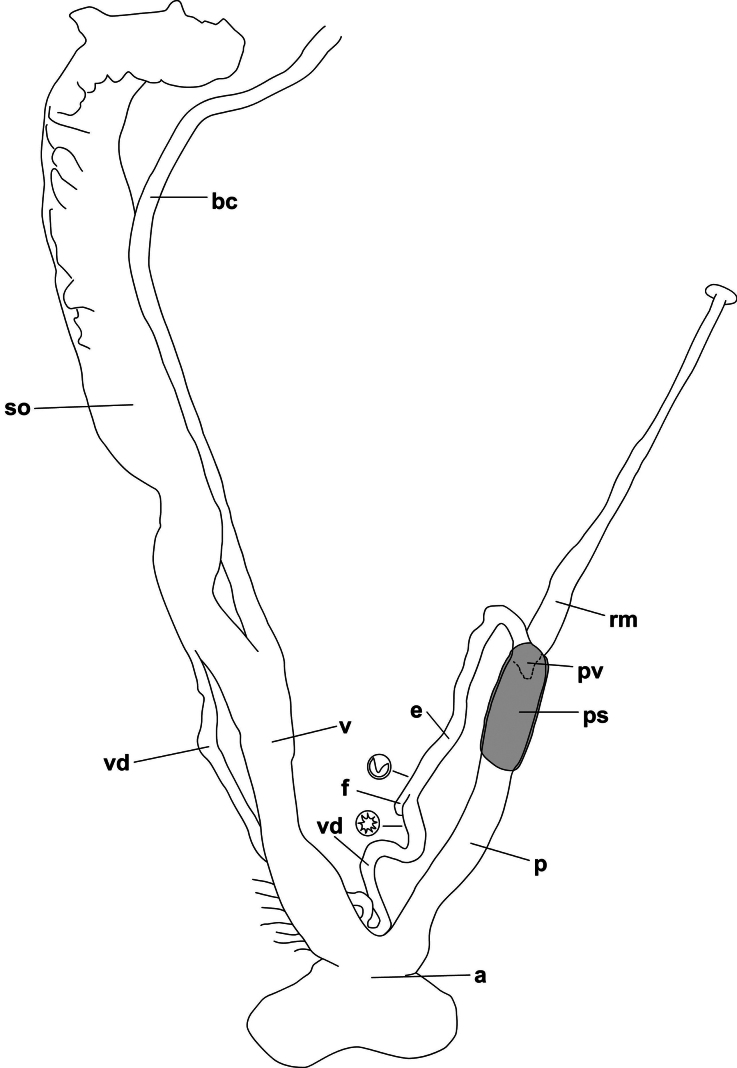
Reproductive anatomy of *Fansipanica milae* gen. et sp. nov. (holotype). Abbreviations: a: atrium; bc: stalk of bursa copulatrix; e: epiphallus; f: flagellum; p: penis; ps: penial sheath; pv: penial verge; rm: retractor muscle; so: spermoviduct; v: vagina; vd: vas deferens.

**Figure 4.**
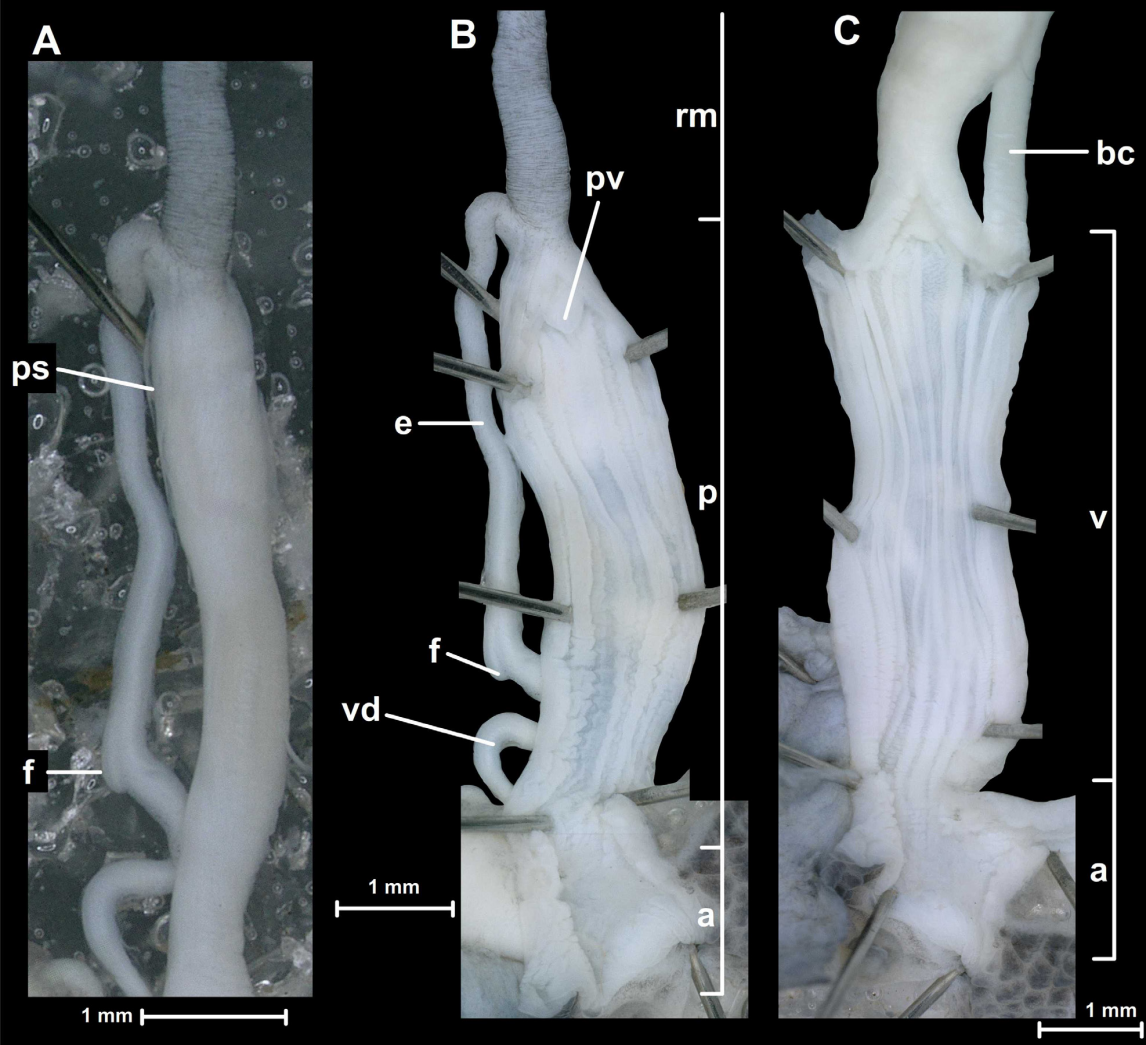
Penis and inner structure of the genital organs of *Fansipanica milae* gen. et sp. nov. (holotype). **A**. Penis, epiphallus and flagellum; **B**. Inner wall of the penis; **C**. Inner wall of the vagina. Abbreviations: a: atrium; bc: stalk of bursa copulatrix; e: epiphallus; f: flagellum; p: penis; ps: penial sheath; pv: penial verge; rm: retractor muscle; v: vagina; vd: vas deferens.

##### Description.

***Body***. Body surface granular, uniformly dark grey coloured, including long eye tentacles. Area around genital pore smoother and paler. The sole is also paler, whitish-yellowish.

***Shell***. The shell of the living animal is dark brown coloured with brown-reddish hue, in preserved specimens pale reddish brown. The shell is fragile, medium sized, globose, slightly wider than high, the subsutural furrow is absent, the body whorl is rounded. The 4.25–4.5 regularly increasing whorls are separated by a shallow suture. The protoconch is consisting of 1.25–1.5 whorls, and bears dense, oval scales. This sculpture abruptly changes to the teleoconch sculpture at the protoconch-teleoconch boundary. The teleoconch is evenly covered by widely spaced hairs (where absent: raised, papilliform hair scars). The hairs are permanent on the entire shell. The hair density is higher on the ventral side near the umbilical area than on the dorsal side and the periphery of the last whorl. The aperture is semilunar, slightly oblique to shell axis (prosocline). The parietal callus is absent, the parietal region is only indicated with an additional thin, transparent calcareous layer. The peristome is pinkish-reddish, slightly expanded and reflected only near the umbilical area. The umbilicus is extremely narrow, almost covered by the reflected peristome.

##### Measurements.

D = 11.9–12.8 mm, H = 9.4–9.8 mm (*n* = 3).

##### Genital organs.

The atrium is short. The penis is long, nearly cylindrical, its proximal (“apical”) third is covered with a thin penial sheath. The inner wall of penis is covered by irregular, slightly serrate longitudinal wrinkles, which gradually becoming stronger (more elevated) and more regular from the atrium towards the proximal end, where their number is five. The penial verge is small, conical, opening along a slit that ends close to its tip. The thick penial retractor muscle inserts at the penis-epiphallus junction. The epiphallus is slender, slightly shorter than the penis, internally with a single, elevated fold. A small, blunt flagellum marks the boundary between epiphallus and vas deferens. The vas deferens is only slightly more slender than the epiphallus. The vagina is slightly shorter than the penis, its distal part (closer to the atrium) is attached to the body wall with numerous fibres. The inner wall of the vagina bears numerous (~ 10) slender folds that are variable in width. The folds (mostly their distal parts) are slightly serrate, and the inner wall of the proximal part of the vagina (closer to the spermoviduct) is finely sculptured by a maze-like structure. The stalk of the bursa copulatrix is very long (> 2× longer than the penis), approximately as wide as the epiphallus. The bursa was torn off when the animal was pulled out of its shell, therefore its morphology could not be examined.

##### Differential diagnosis.

The most similar species is *F. gereti* (Bavay & Dautzenberg, 1900), comb. nov. due to the similar size (although it is slightly larger), the shape of the shell and the (nearly) closed umbilicus. However, the new species has much denser hairs on the entire shell surface and has a darker peristome.

##### Etymology.

The species is named after Ivailo Dedov’s wife Mila Taseva, for her support over the years and patience during the long field trips.

##### Habitat.

Fansipan Mountain and the adjacent areas in Lao Cai Province (western part of the Hoang Lien Mountains) are a very unique region in northern Vietnam, where a large area of closed canopy primary forest in a mountain habitat persists. Nowadays, due to excessive utilisation, the tropical rainforest is only maintained in disconnected patches. The total number of species that can still be described makes the area rich (Sobey 1997). Hoang Lien National Park is located in the Hoang Lien Mountain Range with Fansipan Peak, which is considered the highest mountain in Vietnam, at 3143 m a.s.l. (Nguyen et al. 2022). The mollusc fauna of this region is almost unknown (Páll-Gergely et al. 2015).

*Fansipanica milae* Páll-Gergely & Dedov, gen. et sp. nov. was found at an altitude of ca 3040 m. The topography here is diversified: the mountain chains alternate with steep slopes and some relatively flat areas appearing like soil hills. The forest in the area of peak was protected from human activity by the long and high slopes, so that it appeared in pristine condition. At this altitude, in addition to the species from the families of Fagaceae and Lauraceae, Ericaceae (*Rhododendron*) was present as well as Theaceae (*Ternstroemia*) and Platanaceae (*Platanus*). On lower slopes and relatively flat places, Fagaceae, Lauraceae, Platanaceae, Sterculiaceae, Elaeocarpaceae, Magnoliaceae, Araliaceae, Aceraceae, Betulaceae, Ericaceae, Theaceae, Rhodoleiaceae, Cupressaceae (young trees), Verbenaceae, Rosaceae, Juglandaceae, and Aquifoliaceae dominate. One specific characteristic of this mountain assemblage is the bamboo forest (cf. *Arundinaria*) under the canopy. On the mountain chains and steep slopes, Theaceae, Ericaceae, Magnoliaceae, Rhodoleiaceae, Lauraceae, Cupressaceae, Aceraceae, Betulaceae, and Rosaceae families are present. The epiphytic plants include Orchidaceae, Lycopodiaceae, Polypodiaceae, Aspleniaceae, Davalliaceae, Hymenophyllaceae, Vittariaceae, Lepidopteridaceae, Gesneriaceae, Ericaceae, Bryophyta, and also Rosaceae and Araliaceae (Sobey 1997).

#### Fansipanica gereti

Taxon classification

Animalia

StylommatophoraCamaenidae

(Bavay & Dautzenberg, 1900) comb. nov.

4E551E6A-E89E-5C9D-83E8-40D5EE9C141C

Fig. 5

Helix (Chloritis) gereti Bavay & Dautzenberg, 1900: 112.
Helix (Chloritis) gereti : Bavay and Dautzenberg 1900: 442, pl. IX, figs 7–9.
Chloritis gereti : Fischer and Dautzenberg 1904: 11.
Chloritis (Trichochloritis) gereti : Gude 1906: 116.
Chloritis gereti : Richardson 1985: 98.
Trichochloritis gereti : Schileyko 2011: 47.

##### Type locality.

“Bac-Kan et Phi-Mi” [Bãc Can: 22°08'N, 105°49'E, see Schileyko 2011, Phi Mi could not be found on the map].

**Figure 5.**
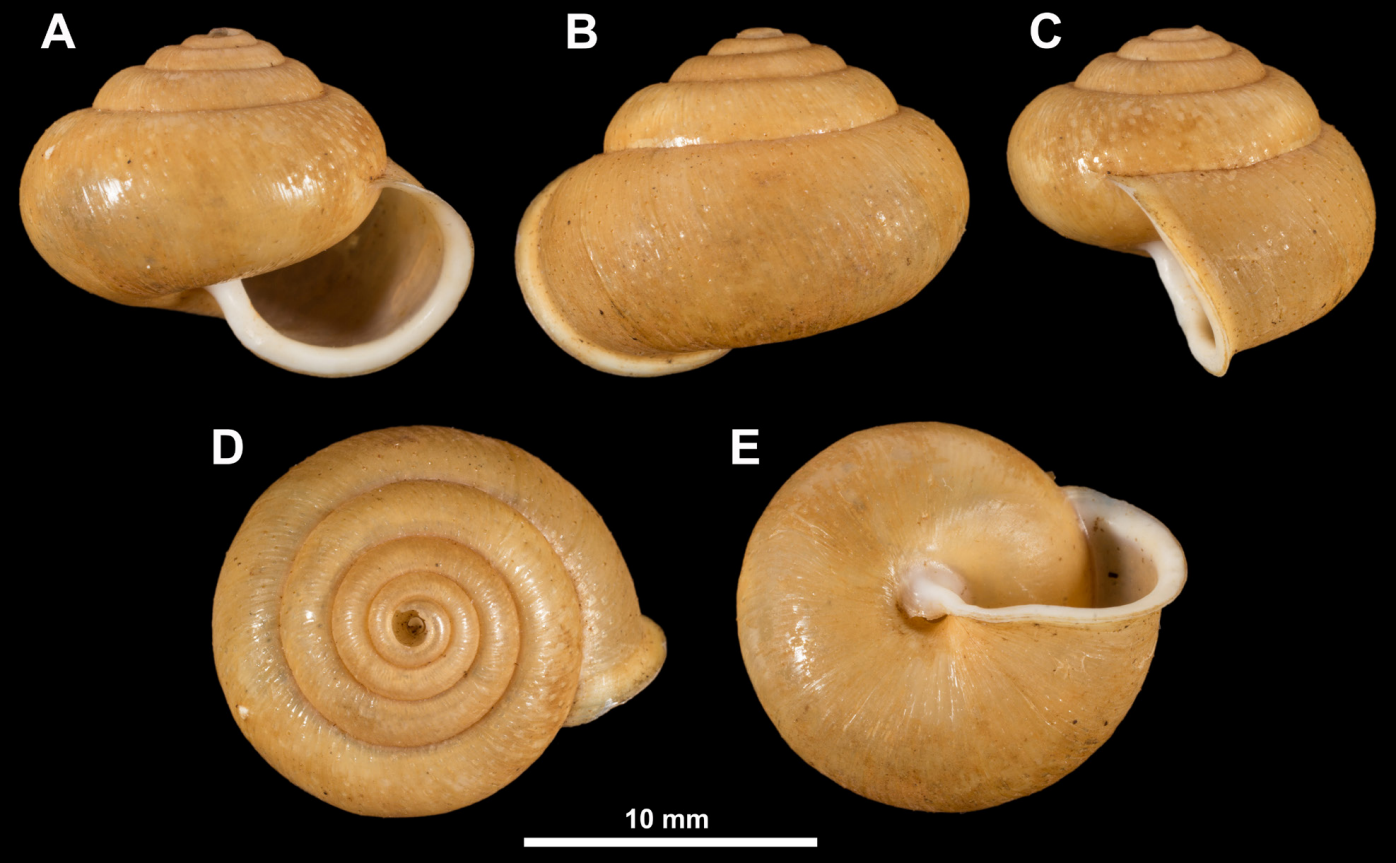
Syntype of *Fansipanica* (?) *gereti* (Bavay & Dautzenberg, 1900) (MNHN-IM-2000-1900). **A**. Apertural view; **B**. View opposite of the aperture; **C**. Lateral view; **D**. Dorsal view; **E**. Ventral view.

##### Type material.

***Syntype***. • Bac Kan et Phi-Mi, leg. Messager, MNHN-IM-2000-1900 (1 specimen) Fig. 5.

##### Additional material.

• Tonkin, Bac Kan, coll. Messager, MNHN-IM-2012-27100 (48 shells) (this is probably the original lot); • Bac-Kan, coll. Denis MNHN-IM-2012-27099 (7 shells); • Tonkin, Bac Kan, coll. Letellier MNHN-IM-2012-27101 (1 shell); • Tonkin, Bac-Kan, coll. Staadt MNHN-IM-2012-27102 (3 shells); • Tonkin, “v. minor”, MNHN-IM-2012-27103 (2 shells); • Tonkin, MNHN-IM-2012-27104 (13 shells); • Tonkin, Salisbury collection, “imaged by H. Taylor (NHM) for North Vietnamese Land Snail Guide Mollusca Section Neg. No. 2050”, NHMUK 20250272 (1 shell).

##### Diagnosis.

A small species with globular shell, closed or nearly closed umbilicus, irregularly wrinkled shell bearing deep, regularly arranged hair scars.

##### Description.

The shell is small, globular, pale brown. The subsutural furrow is absent, the body whorl is rounded. The protoconch consists of 1.5 whorls, and is very finely, irregularly wrinkled and bears hair scars. The teleoconch is finely and irregularly wrinkled, and covered by widely spaced but rather regularly arranged deep hair scars. The hairs are probably not permanent (although no live collected specimens were available). The 4.75–5 whorls are separated by a moderately deep suture. The aperture is semilunar. The peristome is expanded but not reflexed, whitish. The parietal region is glossy, paler than the rest of the shell in some specimens. The umbilicus is very narrow, covered by the reflection of the columellar peristome, in some specimens covered entirely.

##### Anatomy of genital organs.

Unknown.

##### Measurements.

D = 13.7–16.4 mm, H = 10.9–13.0 mm (*n* = 4).

##### Remarks.

This species is included in *Fansipanica* gen. nov. due to its conchological similarities with its type species. The assignment of *F. gereti* to *Fansipanica* gen. nov. should be considered provisional and based on the only information available at the moment, i.e., the conchological similarity.

#### Ducanhia

Taxon classification

Animalia

StylommatophoraCamaenidae

Páll-Gergely gen. nov.

4EDC2960-5E11-5A5E-B90D-3EA3A687B765

https://zoobank.org/9E4AE76C-2DE1-4101-9284-087EA7C8A5AF

##### Type species.

*Helix balansai* Morlet, 1886.

##### Diagnosis.

Shell relatively large, dorsal side nearly flat, umbilicus only slightly open, entire shell covered with hairs. Small penial verge present with central opening; flagellum present, penial caecum absent; retractor muscle inserts near the distal end of the epiphallus; stalk of bursa copulatrix very short.

##### Differential diagnosis.

The anatomical traits differentiate this new genus from all Southeast Asian *Chloritis*-like genera. *Bellatrachia* has no penial verge and has a thickened base of the bursa copulatrix (Schileyko 2018; Páll-Gergely and Neubert 2019). *Bouchetcamaena* has a much more elongated, pointed flagellum, and lacks a penial verge (Páll-Gergely et al. 2022). *Trichochloritis* has a penial caecum (Páll-Gergely and Neubert 2019). Continental *Chloritis* species have a pointed flagellum and a less muscular, thinner walled penis (Sutcharit and Panha 2010; Páll-Gergely et al. 2020). Moreover, all mentioned genera have a longer stalk of the bursa copulatrix.

##### Etymology.

this new genus is dedicated to and named after Duc Anh Nguyen, specialist of millipedes, a much-valued friend of the first author. Genus feminine.

##### Remarks.

The key traits of this new genus are the short bursa copulatrix, and the complicated inner structure of the penis. Namely, distally (i.e., in direction of the atrium) from the small penial verge, there is a sphincter-like region between the penial verge and the most distal (basal) part of the penis. A similar penial structure was described in *Planispira* H. Beck, 1837 (see Schileyko 2003). However, the latter genus inhabits a different biogeographical region (New Guinea and Moluccas) and *Planispira* possesses a long stalk of the bursa copulatrix.

Recently, *Helix balansai* was classified in the genus *Trachia* Martens, 1860. Unfortunately, the anatomy of type species of *Trachia* (i.e., *Helix asperella* L. Pfeiffer, 1846) is unknown. *Helix asperella* was described from India (type locality: “Bithonia in ripa Gangis”) and was subsequently reported from several localities in central and southern India (see Gude 1914; Ramakrishna Mitra and Dey 2010), but not from the eastern Himalayas, which would have biogeographical connections with Southeast Asia. Schileyko (2018) assigned several species to *Trachia* from India, Myanmar, Sri Lanka, and the Andaman Islands based on conchological similarities, including *Trachia delibrata* (W.H. Benson, 1836), the only species with known anatomical traits. The latter species, however, is known from the western Himalayas (type locality: “North-East frontier of Bengal”), and was classified in *Bouchetcamaena* based on anatomical similarities with other *Bouchetcamaena* species (Páll-Gergely et al. 2022). Therefore, it seems that *Trachia* is a genus restricted to central and southern India and maybe Sri Lanka and the Andaman Islands, but species east of the Himalayas would belong to other genera.

#### Ducanhia balansai

Taxon classification

Animalia

StylommatophoraCamaenidae

(Morlet, 1886)

8DE70235-C63A-5418-A03A-B360F95EA606

Figs 6, 7, 8, 9

Helix balansai Morlet, 1886: 7.
Helix balansai : Morlet 1887: 258, 270, 271, pl. 12, figs 4, 4a, 4b.
Helix balansai : Dautzenberg and d’Hamonville 1887: 218. (Rochers de marble du Nuy-Dong-Nay [marble rocks of Dong Nai Province])
Helix (Trachia) balansai : Fischer 1891: 26.
Chloritis balansai : Fischer and Dautzenberg 1904: 11.
Helix (Chloritis) balansai var. *cincta*: Dautzenberg and Fischer: 1905: 90, pl. 3, figs 5–9.
Chloritis (Trichochloritis) balansai : Gude 1906: 116.
Chloritis (Trichochloritis) balansai var. *cincta*: Gude 1906: 116.
Chloritis balansai : Richardson 1985: 86.
Trachia balansai : Schileyko 2011: 45.

##### Type locality.

Not stated (Tonkin – from title) (*H. balansai*); Ile Krieu, Archipel des Faï-Tsi-Long, Tonkin [Krieu Island, Ha Long Provincial, Quang Ninh Province, Vietnam] (*H. balansai* var. *cincta*).

**Figure 6.**
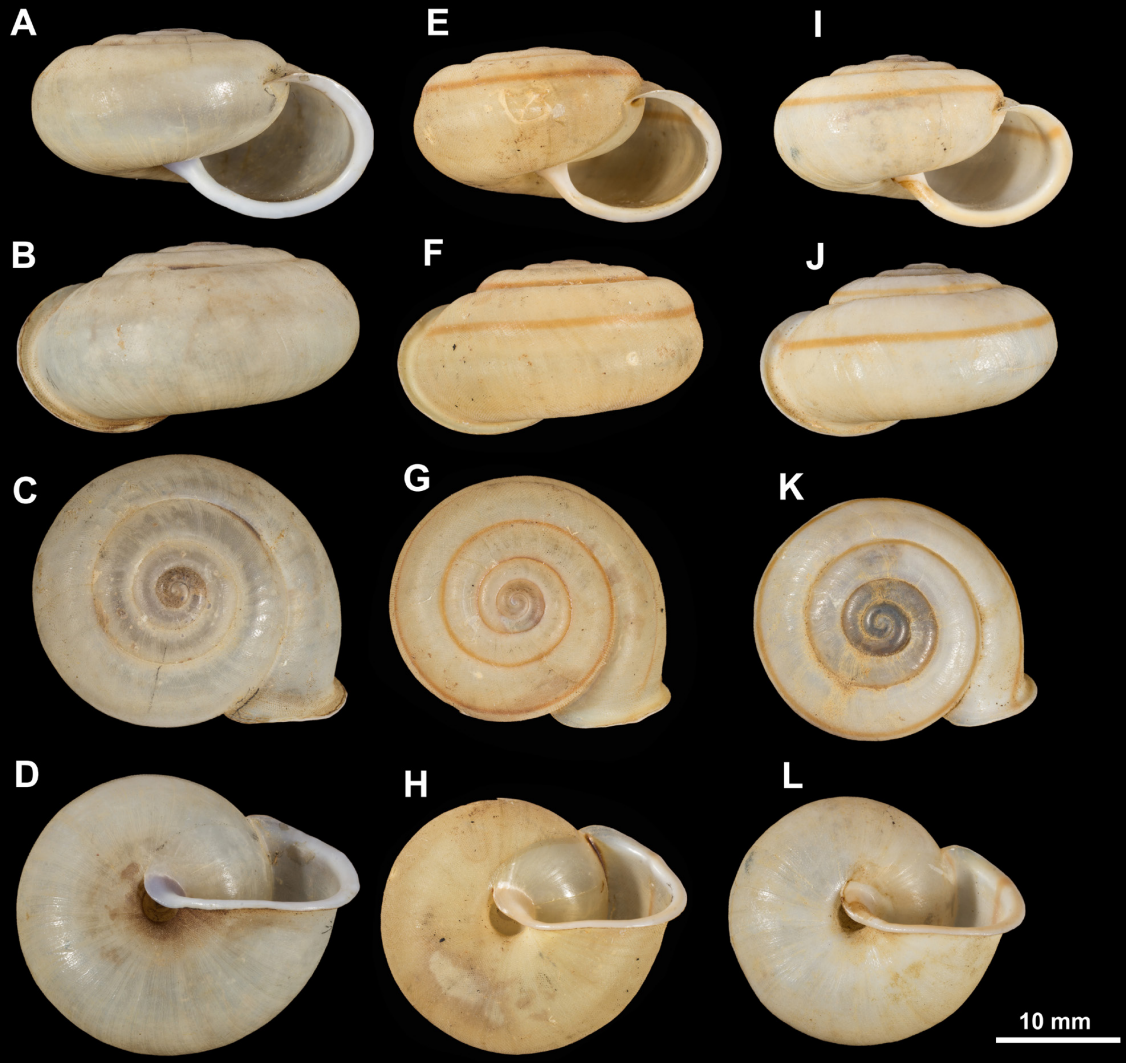
Shells of *Ducanhia balansai* (Morlet, 1886). **A–D**. Syntype of *Helix balansai* (MNHN-IM-2000-2078); **E–H**. Syntype of *Helix balansai* var. *cincta* (MNHN-IM-2000-2077, spec. 1); **I–L**. Syntype of *Helix balansai* var. *cincta* (MNHN-IM-2000-2077, spec. 2).

**Figure 7.**
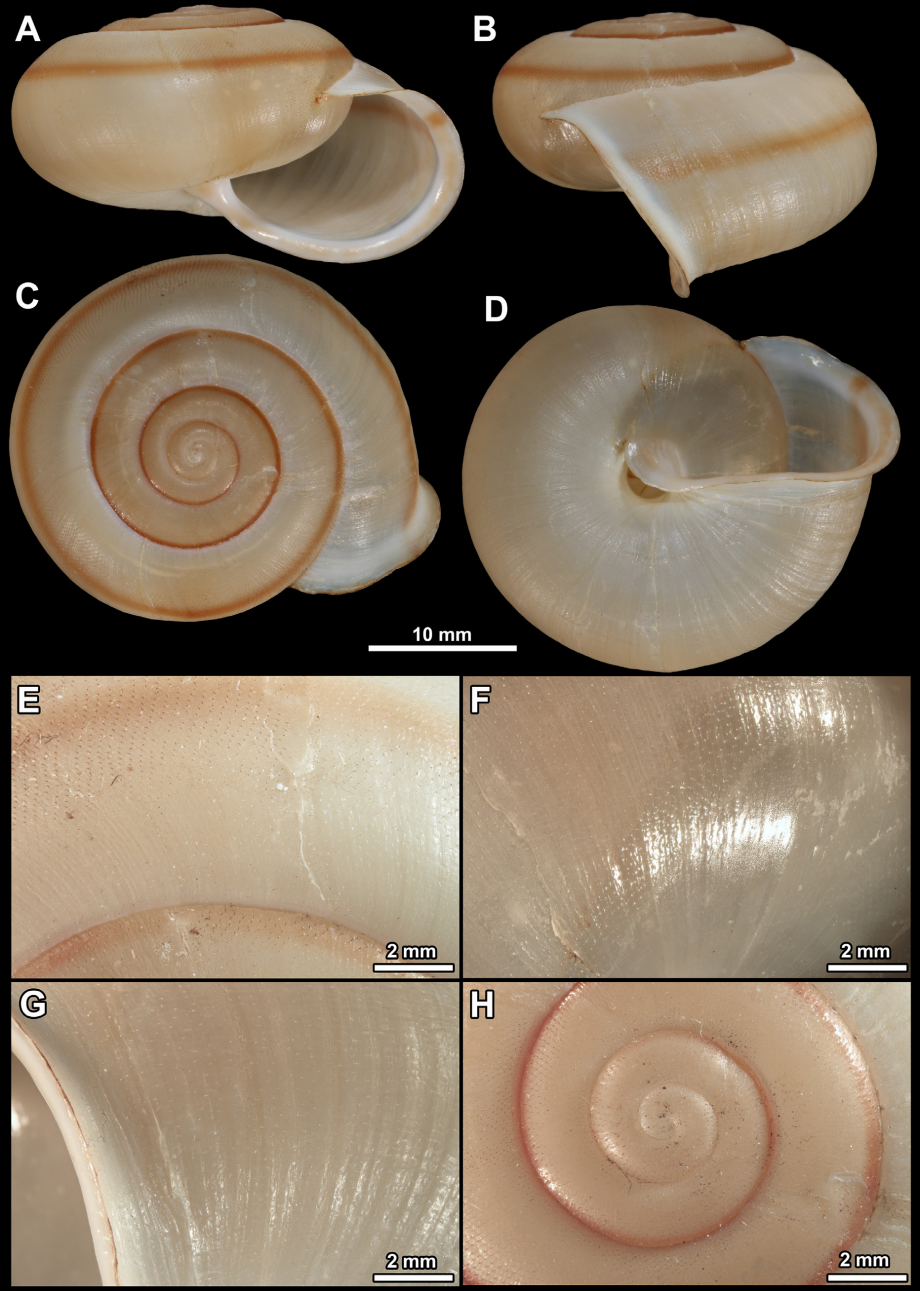
Anatomically examined specimen of *Ducanhia balansai* (Morlet, 1886). For the positions of the sculpture photos see Páll-Gergely et al. (2023: fig. 1). **A**. Apertural view; **B**. Lateral view; **C**. Dorsal view; **D**. Ventral view; **E**. Dorsal side, last and penultimate whorls; **F**. Parietal callus area; **G**. Ventral side of the last whorl just behind the peristome; **H**. Protoconch and first teleoconch whorls.

**Figure 8.**
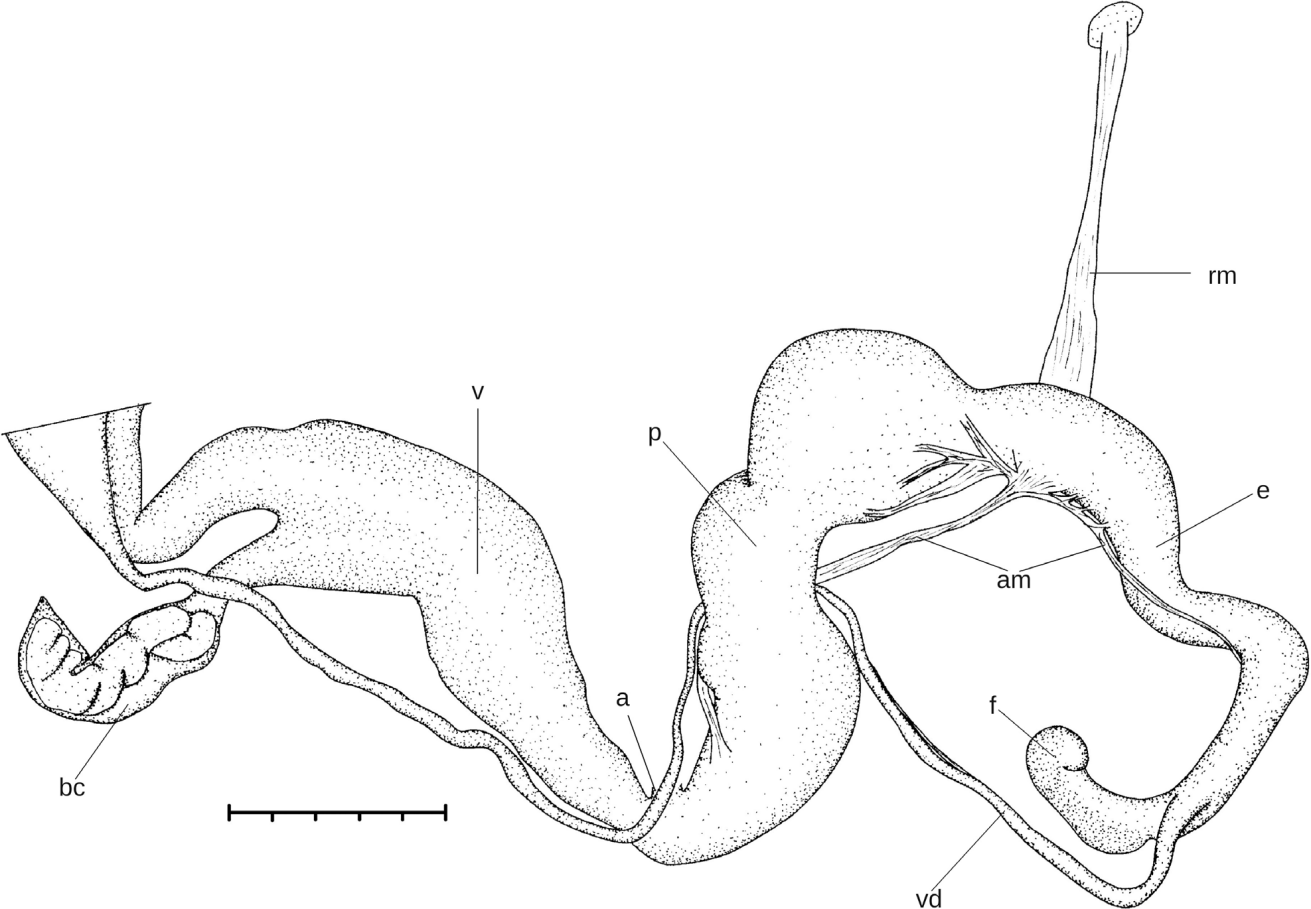
Genitalia of *Ducanhia balansai* (Morlet, 1886). Abbreviations: a: atrium; am – additional muscle; bc: bursa copulatrix; e: epiphallus; f: flagellum; p: penis; rm: retractor muscle; v: vagina; vd: vas deferens.

**Figure 9.**
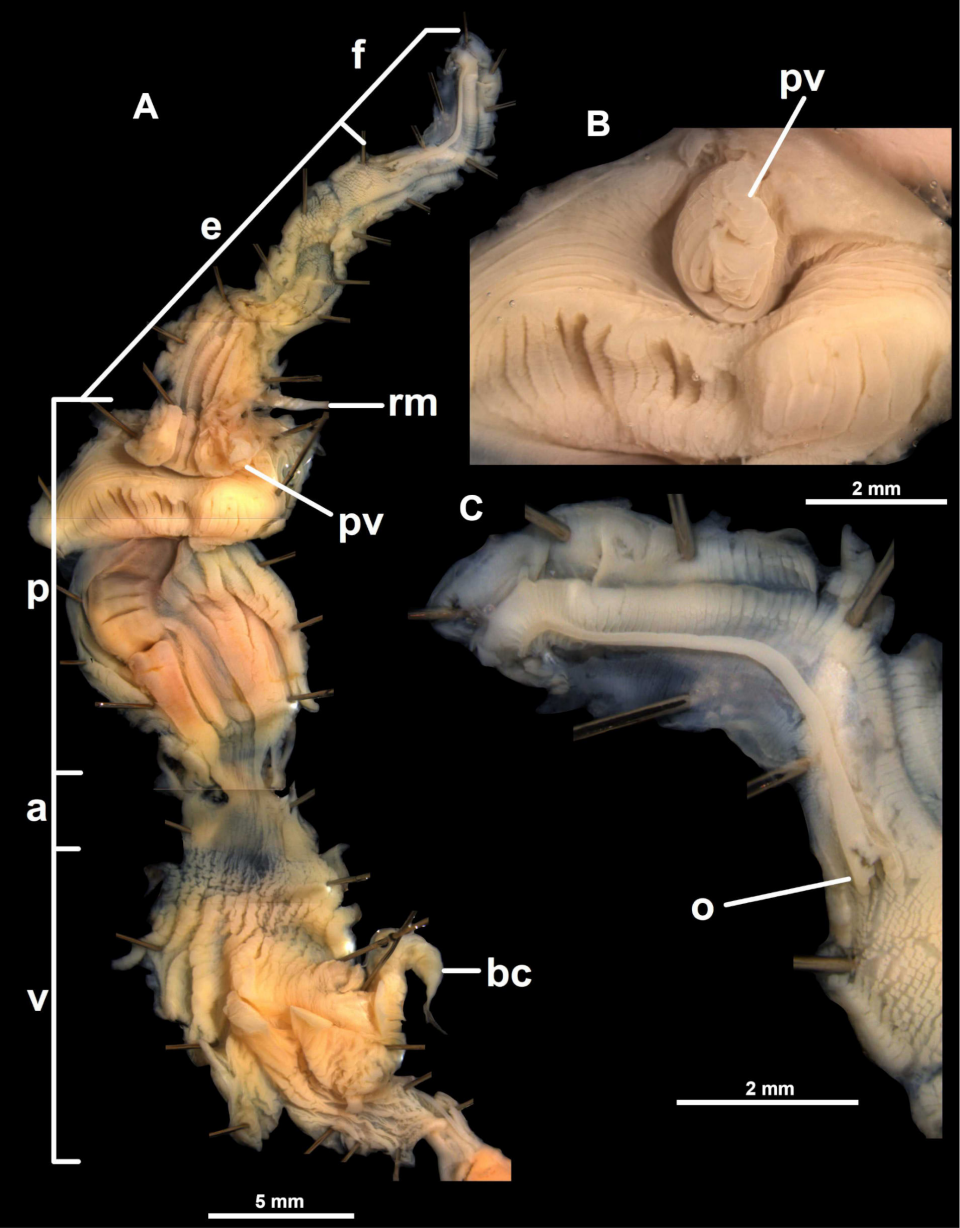
Inner structure of the genital organs of *Ducanhia balansai* (Morlet, 1886). **A**. Entire genitalia from vagina to flagellum; **B**. Penial verge; **C**. Flagellum. Abbreviations: a: atrium; bc: bursa copulatrix; e: epiphallus; f: flagellum; o: opening of the elevated fold inside the flagellum; p: penis; pv: penial verge; rm: retractor muscle; v: vagina.

##### Type material examined.

***Syntypes*** • MNHN-IM-2000-2078 (1 syntype of *H. balansai*); • MNHN-IM-2000-2077 (2 syntypes *H. balansai* var. *cincta*).

##### Additional material.

• Tonkin, ex Balansa 1887 MNHN-IM-2013-49807 (1 shell); • Tonkin, coll. Blaise MNHN-IM-2013-49808 (9 shells); • Tonkin, Ile Krieu, coll. Blaise, MNHN-IM-2013-49809 (5 adult + 1 juvenile shells); • Tonkin, Ile Krieu, archipel des Fai-Tsi-Long, coll. Blaise, MNHN-IM-2013-49811 (s juvenile shells); • Tonkin, coll. Jousseaume MNHN-IM-2013-49810 (2 shells); • Tonkin, leg. Abbé Vathelet, MNHN-IM-2013-49812 (6 shells); • Baie d’Along, leg. Abbé Vathelet 1887, MNHN-IM-2013-49813 (3 shells); • Vietnam, Quang Ninh Prov., Halong Bay Area, unnamed island 1.8 km W of S point Cong Tai Isl., steep limestone slope bordering beach, dense vegetation, handpicked + soil sample, 20°52.29N, 107°18.15E, J.J. Vermeulen & A.J. Whitten leg., 3 Oct. 1998, ex coll. Vermeulen 6455, NHMUK 19991421 (2 complete + 1 damaged shells) “*Chloritis balansai cincta*”; • Vietnam, Cat Ba Island, Cat Ba N.P., Hai Phong City, near pass in front of May Bau, 20.7969°N, 107.0070°E, ca 97 m, leg Otani, J.U., 22.11.2007, IEBR_LS_Ducanhia001H.

##### Diagnosis.

Shell large, thin, bright yellow, almost flat, with a subsutural furrow; hairs cover the complete shell, umbilicus partly to almost completely covered by hair scars or bristles.

##### Description.

The shell is large, thin, depressed, with only a slightly elevated spire and somewhat domed apical part. The body whorl is somewhat expanded with a clearly marked subsutural furrow visible on the whole last whorl. The shell colour is pale greyish to yellow, a red spiral band may run above the furrow. The protoconch consists of 1.75–~2 whorls, completely covered by dense pattern of hair scars. The teleoconch also beats hairs (in fresh shells) or hair scars (weathered shells). The 4.25–4.75 whorls are separated by a rather shallow suture. The hairs are short and tend to be permanent. The aperture is semilunar, the reflected peristome reinforced by a thick white or even reddish lip. The parietal region is with an inconspicuous additional layer which is not necessarily paler than the rest of the shell, but glossy. The umbilicus is slightly funnel-shaped and partly to almost completely concealed by the reflection of the columella.

##### Measurements.

D = 23.2–24.5 mm, H = 13.0–13.6 mm (*n* = 2: MNHN-IM-2013-49808).

##### Description of the genital organs.

The right ommatophoral retractor crosses the penis and the vagina. The atrium is very short. The penis is relatively long, consists of a spindle-shaped distal and a thickened, globular proximal portion. The inner wall of the distal part has irregular longitudinal, thick folds; the thickest longitudinal fold bears some transversal wrinkles. The proximal penial portion internally has a strongly thickened sphincter-like structure with one side being convex, and the other concave. These two sides fit exactly to each other. Just above of this muscular sphincter-like part there is a small, papilla-like penial verge having central opening. The long epiphallus consists of a thicker distal, and a slender proximal portion. The boundary between these two parts is transitional, not abrupt. The thicker part internally with four longitudinal, slightly serrate folds. The thinner portion of the epiphallus internally with small rhomboid papillae. The retractor muscle attaches on the diaphragm and inserts near the distal end of the epiphallus. There is an additional, weaker muscle, which is divided into several branches inserting on the proximal part of the penis and the distal part of the epiphallus. The flagellum is very short, with a curved tip. The flagellum is internally with broad, low, ribbed longitudinal folds, and an elevated, slender longitudinal fold starting from the point where vas deferens originates. The vas-deferens is overall slender. The vagina is approximately as long as the penis, its distal end is the thinnest and it gradually tapers towards the spermoviduct. The distal part of the vagina (i.e., closer to the atrium) internally bears strongly serrated longitudinal folds. In proximal direction (i.e., towards the spermoviduct) these folds become less serrated and weaker. At the proximal end of the vagina there are free, elevated lobes internally. The bursa copulatrix is conspicuously short, thick, and with a rather pointed end. An amorphous spermatophore was found inside the bursa.

##### Remarks.

Inkhavilay et al. (2019: fig. 48D) reported this species from Laos. However, the shell they figured has a wider umbilicus, and lacks the subsutural furrow characteristic for typical *Helix balansai* shells. Moreover, that shell had multiple reddish bands on the body whorl, suggesting that it may belong to *Burmochloritis* (see Páll-Gergely et al. 2023) instead of conspecific with *H. balansai*.

The red spiral may be present or absent even in the same populations, so it does not justify a separation of a subspecies. The degree of coverage of the umbilicus varies from only a very small proportion concealed to almost completely reflected.

#### Vinatachea

Taxon classification

Animalia

StylommatophoraCamaenidae

Thach, 2025

2B2B067D-F08D-5CF9-950B-B06CF7396DF2

Vinatachea Thach, 2025: 83.

##### Type species.

*Vinatachea thienanae* Thach, 2025 (by original designation).

##### Diagnosis.

The short, blunt penis, the epiphallus, which is very slender when connected to the penis, the absence of the flagellum and a penial caecum and the strongly thickened vas deferens distinguishes this genus from all other camaenid genera in Southeast Asia.

##### Remarks.

The anatomy of the type species is unknown. *Vinatachea porcellana* sp. nov. is classified in this genus provisionally, due to the similar shell size and shape, and the geographic proximity.

#### Vinatachea (?) porcellana

Taxon classification

Animalia

StylommatophoraCamaenidae

 Páll-Gergely sp. nov.

FA909BD6-E6A3-59E8-A13C-F69CD632F1E5

https://zoobank.org/4C451B41-8AFF-48E4-A6FE-BB4B96D8FAF0

Figs 10, 11, 12, 13

##### Type material.

***Holotype*** • **Vietnam**, Nghe An Province, Con Cuong District, Anh Son District, Hoi Son commune, Pu Huong Nature Reserve, 18.951979°N, 105.043838°E, ca 28 m, J. U. Otani leg., 10 May 2008 (empty shell and body in ethanol), IEBR_LS_Ngheania001H.

**Figure 10.**
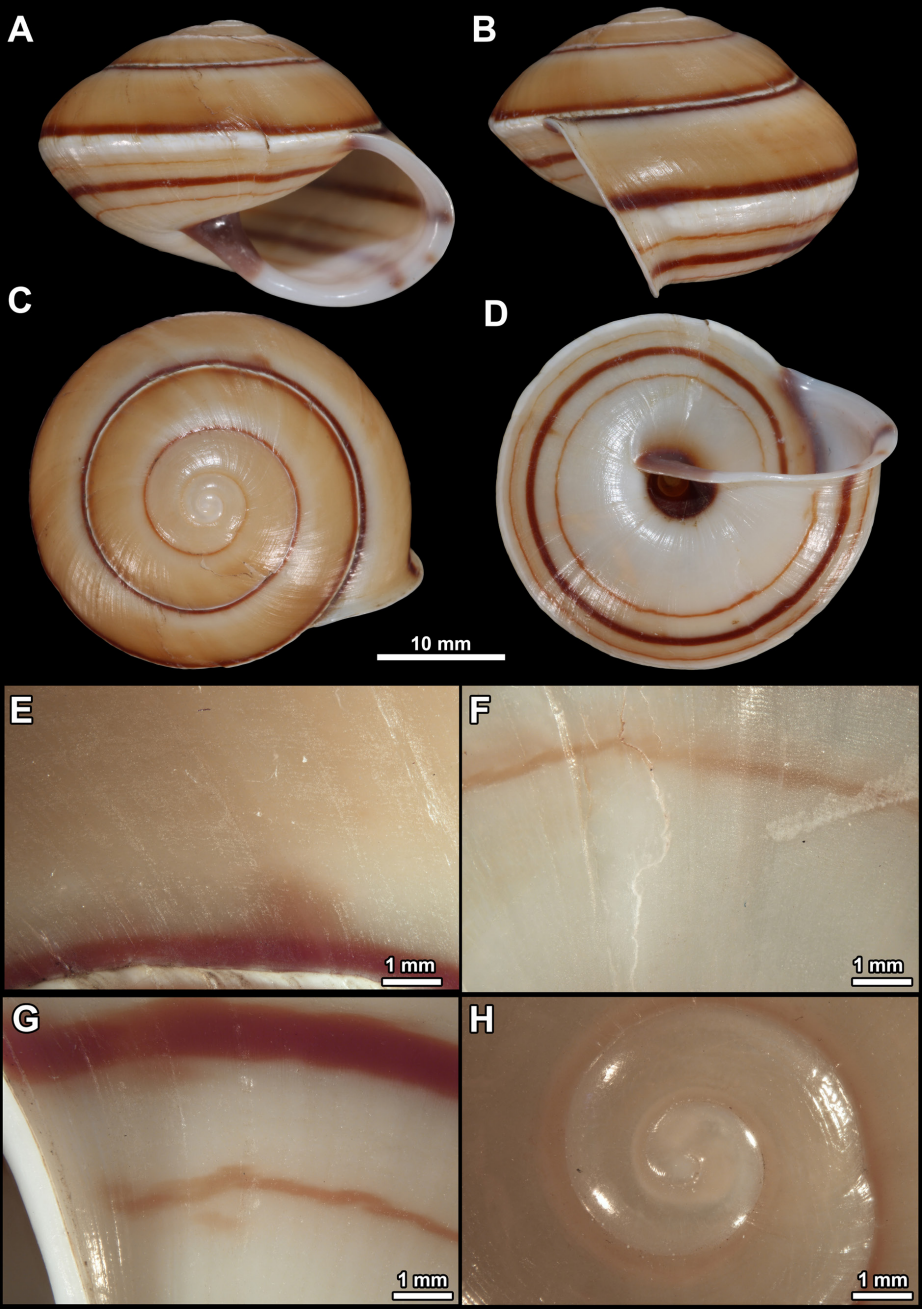
Holotype of *Vinatachea porcellana* sp. nov. For the positions of the sculpture photos see Páll-Gergely et al. (2023: fig. 1). **A**. Apertural view; **B**. Lateral view; **C**. Dorsal view; **D**. Ventral view; **E**. Dorsal side, last whorl; **F**. Parietal callus area; **G**. Ventral side of the last whorl just behind the peristome; **H**. Protoconch and first teleoconch whorls.

**Figure 11.**
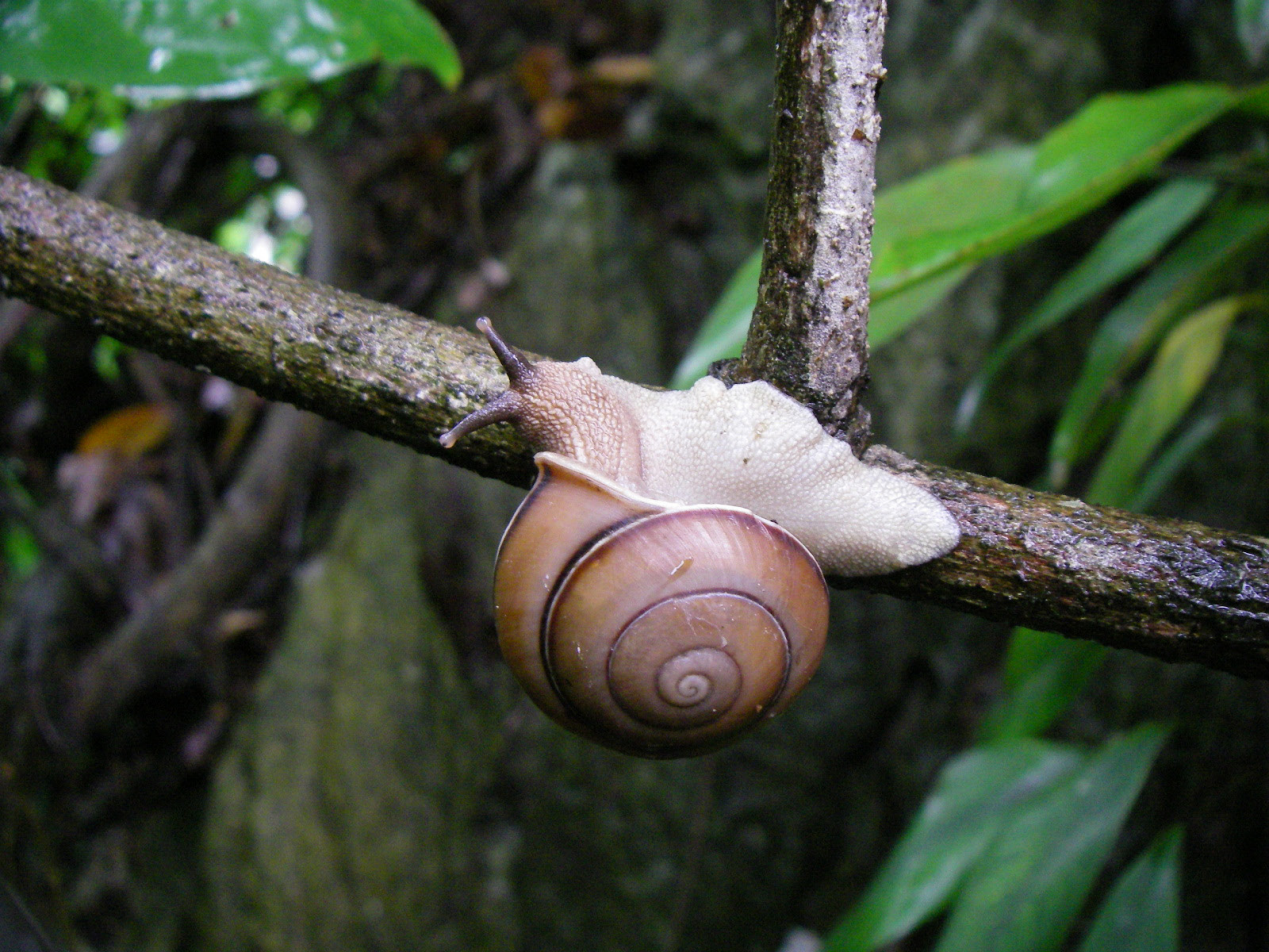
Living holotype of *Vinatachea porcellana* sp. nov. Photo: J. U. Otani.

**Figure 12.**
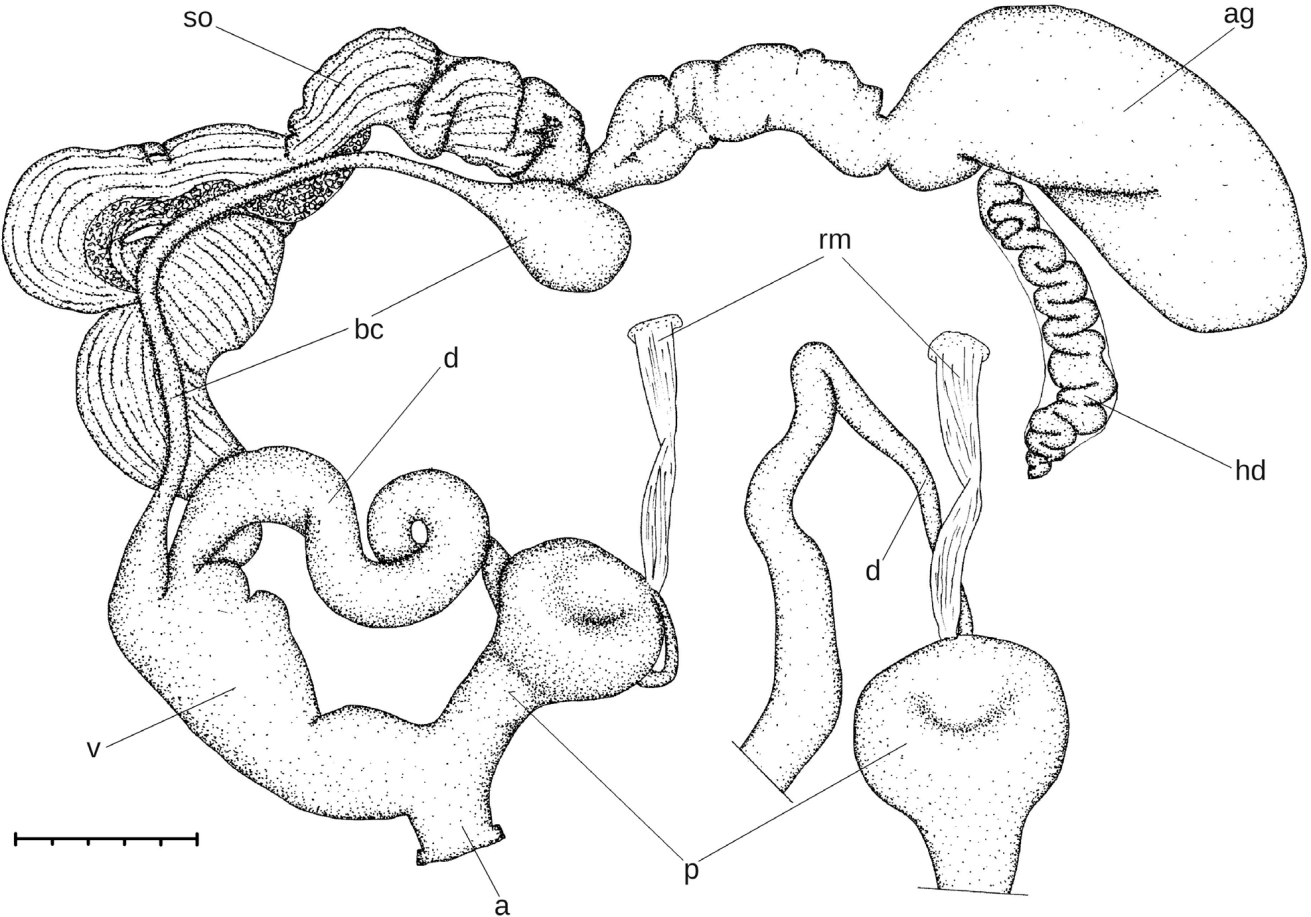
Reproductive anatomy of *Vinatachea porcellana* sp. nov. (holotype). Abbreviations: a: atrium; ag: albumen gland; bc: bursa copulatrix; d – duct (probably epiphallus + vas deferens); hd: hermaphrodite duct; p: penis; rm: retractor muscle; so: spermoviduct; v: vagina.

**Figure 13.**
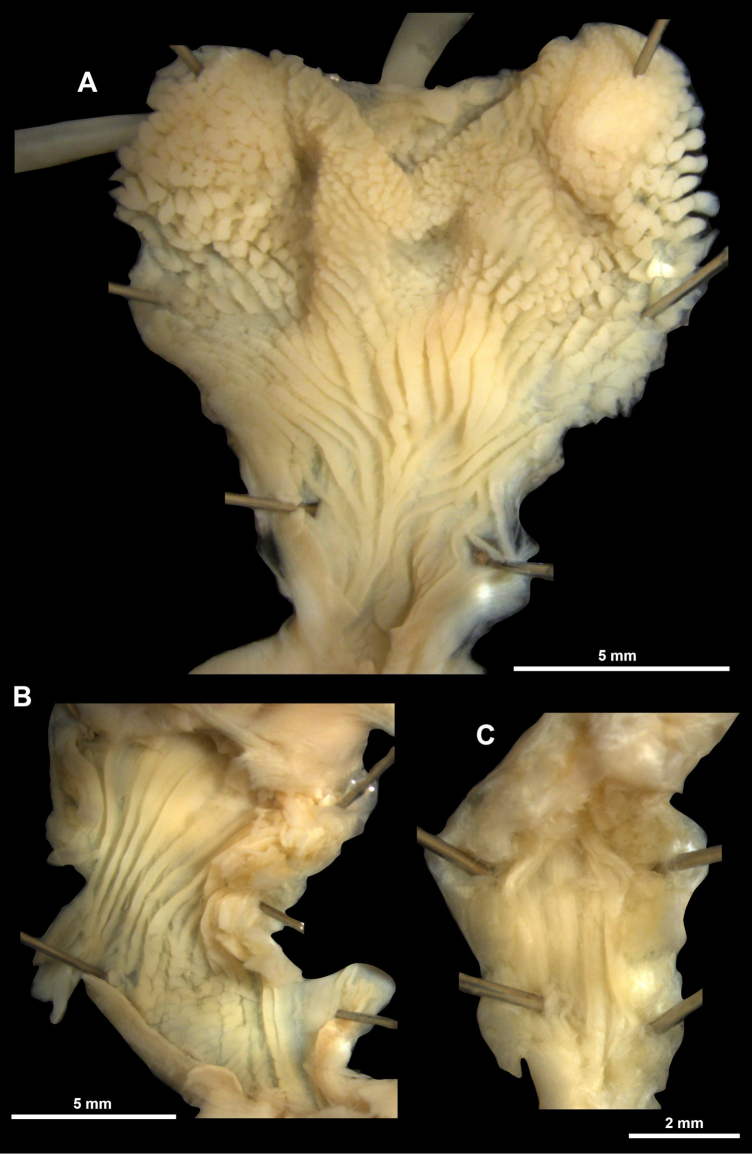
Inner structure of the reproductive anatomy of *Vinatachea porcellana* sp. nov. (holotype). **A**. Penis; **B**. Vagina; **C**. End of vas deferens.

##### Description.

***Shell***. The shell is large, thick walled, overall glossy, nearly smooth. The shell shape is helicoid, with a blunt keel on the periphery. The shell colour is complex. The basic colour is ochre, and there is a slender, dark brown belt running just above the keel. Below the brown belt, there is a white belt, which is ~ 2× thicker than the brown one. On the dorsal side, there is a thin, white belt above the suture, and a thin brown belt below the suture. The base colour of the ventral side is slightly paler than that of the dorsal surface. There is a thick (~ 1 mm), dark brown band on the ventral side, with two much more slender brown bands in both directions. The inner side of umbilicus is chocolate brown. The protoconch consists of ~ 1.75 whorls, and is rather glossy, with irregular fine wrinkles and spiral grooves. The sculpture of the teleoconch is dominated by extremely fine spiral grooves and fine growth lines. The entire shell consists of 4.75 whorls separated by a shallow suture. The aperture is semilunar, the parietal side is nearly straight. The peristome is discontinuous, strongly expanded, not reflected, porcelain white in colour, although there are three chocolate brown spiral bands on the ventral side, while the umbilical colour is also visible. The parietal callus is indicated by a transparent, thin calcareous layer, making the surface matte. The umbilicus is narrow, shows all whorls, and is partly covered by the reflected peristome.

##### Measurements.

D = 31.3 mm, H = 20.7 mm (*n* = 1).

##### Description of the genital organs.

The right ommatophoral retractor crosses the penis and the vagina. The atrium is short and thick. The penis is pear-shaped, consists of a slimmer distal (= closer to the genital opening) and a thickened proximal portion. The latter bears a slight central depression. The internally widened part of penis has numerous elongated, small papillae (some of the filiform), which are arranged in longitudinal folds that are converging towards atrium. The penial verge and the flagellum are absent. A slender duct, probably homologous with the epiphallus, starts from thickened part of penis, just from the base of the long and slender retractor muscle. The duct from the penis to the spermoviduct (probably epiphallus + vas deferens) is long, its distal part is slender, tapers until the middle, when it reaches maximum diameter, and remains thick until reaching the spermoviduct. The vagina is strongly developed, overall thick, attached to body wall with several fibres, with externally identifiable, slightly thinner distal, and slightly thicker proximal portion. The thinner part internally bears finely reticulate sculpture, while the inner wall of the thicker part is ornamented by ~ 10 longitudinal folds. The bursa copulatrix has a very slender, long stalk, originating from the insertion point of vas deferens, and a thickened, oval-rounded bursa. The spermoviduct and the albumen gland are very large.

##### Differential diagnosis.

*Vinatachea delsaerdti* (Thach & F. Huber, 2018), comb. nov. is probably the most similar species. However, it has a stronger keel, possesses a single, slender brown belt on its base, and has a much rougher sculpture, most importantly with much more widely-spaced and deeper spiral grooves. The other three species of *Vinatachea* (*V. anhi* Thach, 2025, *V. khoai* Thach, 2026, and *V. thienanae* Thach, 2025) have much more and densely arranged, usually slender spiral bands. *Neotrachia duporti* (Bavay & Dautzenberg, 1909) is larger with a blunter keel, and its colouration is also different (yellowish-greenish base with pale brown or reddish spiral lines). *Camaena gabriellae* (Dautzenberg & d’Hamonville, 1887) has a rougher surface sculpture, a spirally striated, but also rather glossy protoconch, a rounded body whorl, a mostly unicoloured base and a narrower umbilicus. *Camaena marmorivaga* (Mabille, 1889) is larger, has more elevated spire, blunter keel, and open umbilicus. On the other hand, it also belongs to the new genus because it has a smooth, glossy protoconch. *Camaena choboensis* (Mabille, 1889) is much larger than the new species, and has different colouration (greenish base with pale brown stripes).

##### Etymology.

The specific epithet porcellana refers to the porcelain-like glossy surface of the shell.

##### Remarks.

Three Peninsular Malaysian endemic species have been classified in the genus *Kenyirus* Clements & S. K. Tan, 2012. The reproductive anatomy is not known in any of them. *Kenyirus balingensis* S. K. Tan, S. Y. Chan & Foon, 2017 and *K. sheema* Foon, S. K. Tan & Clements, 2015 differ from the type species, *K. sodhii* Clements & S. K. Tan, 2012 considerably, suggesting that they may belong to different genera. *Kenyirus sodhii* is superficially similar to *Vinatachea porcellana*, but it differs in the strongly keeled body whorl, the subtriangular peristome, and the spout-like rostrum. Moreover, according to the original description, the protoconch of *K. sodhii* bears axial striae, while that of *Vinatachea porcellana* has spiral grooves.

#### Vinatachea delsaerdti

Taxon classification

Animalia

StylommatophoraCamaenidae

(Thach & F. Huber, 2018) comb. nov.

38565B8C-1613-50AD-BE5F-47287A89B3CE

Camaena delsaerdti Thach & F. Huber, in Thach, 2018: 67–68, figs 889–892.

##### Remarks.

This species and its subspecies *Camaena delsaerdti melanica* Thach & F. Huber, 2018 are moved to *Vinatachea* due to their similarity with the type species of the genus, *V. porcellana* sp. nov. The holotype of the second subspecies, *C. delsaerdti aurantia* Thach & F. Huber, 2020 is a juvenile, corroded shell, which lacks the important traits for classification; therefore, it is considered a taxon inquirendum.

## Discussion

In the current paper we examined the reproductive anatomy of three species of relatively large, depressed shelled Camaenidae from Vietnam. Among them, two are new species described herein, *Fansipanica milae* sp. nov. and *Vinatachea porcellana* sp. nov., and the third one is a species that was described at the end of the 19^th^ century, *Ducanhia balansai*. When assigning these species to genera based on conchological and anatomical information, first we listed all the camaenid genera of continental SE Asia from comprehensive regional faunal works (Southern Vietnam: Schileyko 2011; Laos: Inkhavilay et al. 2016; Cambodia: Sutcharit et al. 2020), additional recent taxonomic publications, and MolluscaBase (2026). From the latter source, we took all genera of the Camaeninae and the camaenid genera not assigned to any subfamilies. Second, we deleted the genera with species having high spired shells, genera of geographically remote areas (New Guinea, Philippines, Indonesia, Southern India, Indonesia, islands of the Pacific Ocean) the ones with apertural barriers and constrictions, and all Bradybaeninae, which are usually smaller and usually possess a dart sac and mucous glands. As a result, we were left with a list of genera with which our three species could be compared to: *Bellatrachia*, *Bouchetcamaena*, *Burmochloritis*, *Camaena* Albers, 1850, *Chloritis*, *Entadella* Páll-Gergely & Hunyadi, 2016, *Ganesella* W. T. Blanford, 1863, *Neotrachia* Schileyko, 2018, *Planispira*, *Philbouchetia* Thach, 2020, *Satsuma* A. Adams, 1868, *Sinochloritis* M. Wu & Z. Chen, 2019, *Trachia*, *Trichochloritis* Pilsbry, 1891, and *Vinatachea* Thach, 2025. The traits of their reproductive anatomy are compiled in Table 1.

**Table 1.** Reproductive anatomy of Camaeninae from continental Southeast Asia.

	References	Penis	Inner structure of the penis	Epiphallus	Insertion of retractor muscle	Penial caecum	Flagellum	Other traits
*Bellatrachia* Schileyko, 2018	Schileyko (2018), Páll-Gergely and Neubert (2019)	long, cylindrical	with parallel folds	long, cylindrical	penis-epiphallus transition	absent	thick, somewhat swollen, with slender tip	absent
*Bouchetcamaena* Thach, 2018	Páll-Gergely et al. (2022)	long, apically thickened	with parallel folds and a vestigial verge	long, cylindrical	on distal epiphallus	absent	long, slender	absent
*Burmochloritis* Godwin-Austen, 1920	Godwin-Austen (1920), Gergely et al. (2023)	long, thick, cylindrical	with wavy folds, verge absent	long, cylindrical	bounds penis and epiphallus in some distance from their junction	short, pointed	long, slender	a long, cylindrical, organ of unknown homology derives from the wall of vagina
*Camaena* Albers, 1850	Páll-Gergely et al. (2016), Ai et al. (2016), Wang et al. (2020), Chen et al. 2024	relatively short to long, cylindrical	with wavy folds, verge relatively small to long	long, cylindrical	epiphallus	absent	long, slender	absent
“Continental Chloritis”	Sutcharit and Panha (2010), Páll-Gergely et al. (2020)	long, apically thickened	with parallel folds and a large verge	long, cylindrical	on distal epiphallus	absent	medium length, gradually becoming slender	absent
*Ducanhia* gen. nov.	this study	with a spindle-shaped distal and a thickened, globular proximal portion	irregular longitudinal, thick folds, and a papilla-like penial verge	long, with a thicker distal, and a slender proximal portion	near the distal end of the epiphallus	absent	flagellum very short, with a curved ending	bursa copulatrix very short
*Entadella* Páll-Gergely & Hunyadi, 2016	Páll-Gergely et al. (2016), Páll-Gergely and Hunyadi (2019)	relatively short, thick	wavy folds, relatively small to large verge	long, cylindrical	middle of epiphallus	absent	strong, single or double	absent
*Fansipanica* gen. nov.	this study	long, cylindrical	irregular, slightly serrate longitudinal wrinkles, and a small verge	long, slender, cylindrical	penis-epiphallus junction	absent	small, blunt	penial sheath covers proximal (“apical”) third of penis
*Ganesella* W. T. Blanford, 1863	Budha et al. (2012), Sutcharit et al. (2019)	relatively short, thick	wavy folds, relatively small verge	relatively short, cylindrical	epiphallus	absent	relatively large, thick	absent
*Neotrachia* Schileyko, 2018	Schileyko (2018)	short, thick	longitudinal pilasters, broken into series of tubercles	swoolen, ovoid	penis-epiphallus transition	absent	medium length, gradually becoming slender	absent
*Vinatachea* (?) porcellana Páll-Gergely, sp. nov.	this study	short, pear-shaped	numerous elongated (some of the filiform) papillae	slender, tapering towards spermoviduct	penis-epiphallus junction	absent		absent
*Planispira* Beck, 1837	Schileyko (2003)	short, apically thickened	with folds and an ovoid, large verge	long, cylindrical	middle of epiphallus	absent	short, conical	absent
*Satsuma* A. Adams, 1868	Wang et al. (2014), Zhang et al. (2020)	long, cylindrical	with wavy folds, verge absent	long, cylindrical	on distal epiphallus	well-developed, tapering	long to short	absent
*Sinochloritis* M. Wu & Z. Chen, 2019	Wu and Chen 2019	thick, cylindrical	with parallel folds, verge absent	long, cylindrical	on distal epiphallus, and also covers proximal part of penis	large, internally with "peach-shaped epiphallic papilla“ (note that Wu & Chen calls this as part of epihallus)	long, slender, tapering	absent
*Trachia* Martens, 1860 (based on *T. vittata*)	Schileyko (2003)	short, swollen	chaotically arranged pilasters	rather short, thick	incorporated into penial sheath	absent	rather long, gradually becoming slender	penial sheath covers entire penis
*Trichochloritis* Pilsbry, 1891	Collinge (1903), Páll-Gergely and Neubert (2019)	long, apically thickened	unknown	long, cylindrical	on distal epiphallus	moderately long, slender	very short, pointed	absent

Among those genera, the soft anatomy of two genera, *Philbouchetia* and *Vinatachea* is unknown. *Philbouchetia* is a here treated as a junior synonym of *Bellatrachia* because its type species (*Philbouchetia huberi* Thach, 2020) is conchologically very similar to that of Bellatrachia (Helix) condoriana Crosse & P. Fischer, 1863; see Páll-Gergely et al. 2019). *Vinatachea* (?) porcellana Páll-Gergely, sp. nov. conchologically fits in *Vinatachea*. Future studies should confirm that the anatomy of the type species of *Vinatachea* is similar to that of *Vinatachea* (?) porcellana. Nevertheless, the anatomy of *Vinatachea* (?) porcellana is characterised by traits not present in any camaenid genera, and for the time being it is used to characterise the genus *Vinatachea*.

Regarding the other genera, the reproductive anatomy of their type species is known with the exception of *Bouchetcamaena* (*Bouchetcamaena huberi* Thach, 2018) and *Chloritis* (*Helix ungulina* Linnaeus, 1758). Regarding the former genus, the anatomy of *Bouchetcamaena platytropis*, a species similar to *B. huberi* has recently been described, and can be used to characterise *Bouchetcamaena* (Páll-Gergely et al. 2022). The case of *Chloritis* is somewhat more problematic: *Chloritis ungulina* was originally described without stating the type locality (Linnaeus, 1758). Subsequently it turned out to inhabit Seram Island [Ceram] of Indonesia (Zilch 1959–1960, 1966). Species with known anatomies (Rensch, 1937) are recorded from New Ireland and New Britain Islands, east New Guinea, and therefore inhabit a biogeographical region different from the Moluccas, where Seram Island is located. Moreover, the continental species assigned to *Chloritis* based on the sunken spire (Sutcharit and Panha 2010) are probably not related to the type species of *Chloritis* and would deserve a new genus.

With the exception of the above-mentioned genera, the definition of the camaenid genera of mainland Southeast Asia is based on the combination of conchological (shell shape, sculpture, colour patterns) and anatomical characters. However, our knowledge of the reproductive anatomy of these taxa is still poor. For example, *Bouchetcamaena* has 18 species, while the anatomy is only known in two species (Stoliczka 1871; Páll-Gergely et al. 2022; MolluscaBase 2026). In *Burmochloritis* the anatomy is also known in two of the known 16 species (Páll-Gergely et al. 2023). In *Trichochloritis*, one of the largest genera in terms of number of species (29, see MolluscaBase 2026), the anatomy of two species was described in publications written more than a century ago (Stoliczka 1873; Collinge 1903), and those descriptions contain no information on the inner structure of the reproductive organs. The anatomical knowledge covering only a small fraction of the described species limits our understanding of morphological variability within a genus. In the genus *Camaena*, in which the reproductive anatomy is known in multiple species, there is a relatively high degree of intrageneric variability. For example, the penial verge of the type species (*Helix cicatricosa* O. F. Müller, 1774) is large and opens at its end (Páll-Gergely et al. 2016). In other *Camaena* species, the penial verge may have a variable shape and size, often with a lateral opening (Wang et al. 2020). Still, the main traits (presence or absence of a penial verge, flagellum, penial caecum, and their morphology) are consistent within *Camaena*.

The reproductive anatomy of the two new genera described herein differ from all listed genera in qualitative traits and important qualitative differences that justify the distinction of new genera. Namely, *Fansipanica milae* gen. et sp. nov., the type species of *Fansipanica* gen. nov., possesses a penial sheath covering the apical (proximal) part of the penis. No penial sheath with that position is known in the related Camaenidae, although it may be homologous with the fibrous capsule covering a swollen apical part of the penis of *Bouchetcamaena platytropis* (see Páll-Gergely et al. 2022). Furthermore, the epiphallus and the vas deferens of *F. milae* gen. et sp. nov. are of nearly equal diameter, and their boundary is externally only indicated by a tiny flagellum. Regarding *Ducanhia* gen. nov., the short bursa copulatrix of the type species (*Helix balansai*) has not been reported in any other Southeast Asian Camaenidae, and the complicated inner structure of the penis is also unique feature for that genus. The reproductive anatomy of *Vinatachea* (?) porcellana sp. nov. is also characterised by features not known in any other Camaenidae, such as the slim epiphallus, which gradually increases its diameter towards the spermoviduct. In all other Camaenidae the opposite is true: the epiphallus is thicker and the vas deferens is thinner. However, no new genus was described for that species because it is similar to the type species of *Vinatachea* in terms of conchological characters.

The genera described and discussed herein are testable hypotheses of monophyletic groups that predict the distribution of characters (Platnick 1979; Wheeler 2004). It would be certainly possible to classify the species we examined in known genera. However, it would result in unnecessarily “dumping” of species into well-defined genera, and the reduction of diagnostic characters in morphologically defined groups. Such practice will turn existing genera into wastebasket taxa (Páll-Gergely 2017).

## Supplementary Material

XML Treatment for Fansipanica

XML Treatment for Fansipanica milae

XML Treatment for Fansipanica gereti

XML Treatment for Ducanhia

XML Treatment for Ducanhia balansai

XML Treatment for Vinatachea

XML Treatment for Vinatachea (?) porcellana

XML Treatment for Vinatachea delsaerdti

## Additional information

### Artificial Intelligence (AI) use

The authors accept full responsibility for the content of the manuscript, including the disclosure of any use of AI.

No AI tools were used in the preparation of this manuscript.

### Author ORCIDs

B. Páll-Gergely https://orcid.org/0000-0002-6167-7221

V. Gojšina https://orcid.org/0000-0002-0413-9304

I. Dedov https://orcid.org/0000-0003-4445-359X

### Data availability

All of the data that support the findings of this study are available in the main text.
